# Fear Conditioning Downregulates Rac1 Activity in the Basolateral Amygdala Astrocytes to Facilitate the Formation of Fear Memory

**DOI:** 10.3389/fnmol.2017.00396

**Published:** 2017-11-27

**Authors:** Zhaohui Liao, Yezheng Tao, Xiaomu Guo, Deqin Cheng, Feifei Wang, Xing Liu, Lan Ma

**Affiliations:** The State Key Laboratory of Medical Neurobiology, School of Basic Medical Sciences, The Institutes of Brain Science, and The Collaborative Innovation Center for Brain Science, Fudan University, Shanghai, China

**Keywords:** Rac1, astrocyte, structural plasticity, amygdala, fear conditioning, memory formation

## Abstract

Astrocytes are well known to scale synaptic structural and functional plasticity, while the role in learning and memory, such as conditioned fear memory, is poorly elucidated. Here, using pharmacological approach, we find that fluorocitrate (FC) significantly inhibits the acquisition of fear memory, suggesting that astrocyte activity is required for fear memory formation. We further demonstrate that fear conditioning downregulates astrocytic Rac1 activity in basolateral amygdala (BLA) in mice and promotes astrocyte structural plasticity. Ablation of astrocytic Rac1 in BLA promotes fear memory acquisition, while overexpression or constitutive activation of astrocytic Rac1 attenuates fear memory acquisition. Furthermore, temporal activation of Rac1 by photoactivatable Rac1 (Rac1-PA) induces structural alterations in astrocytes and *in vivo* activation of Rac1 in BLA astrocytes during fear conditioning attenuates the formation of fear memory. Taken together, our study demonstrates that fear conditioning-induced suppression of BLA astrocytic Rac1 activity, associated with astrocyte structural plasticity, is required for the formation of conditioned fear memory.

## Introduction

Astrocytes, the most abundant glial cells in the central nervous system, have been generally believed to maintain the homeostasis of metabolism and ionic environment (Allen and Barres, 2009; Weber and Barros, 2015). However, a substantial evidence suggests that they play active roles in the information processing and signal transmission of neural circuits (Volterra and Meldolesi, 2005; Giaume et al., 2010; Clarke and Barres, 2013). For instance, astrocytes secrete various molecules shaping synaptic formation, maturation and even deletion (Christopherson et al., 2005; Allen et al., 2012; Chung et al., 2013), during the development of nervous system. In addition, gliotransmitters released by astrocytes might generate complicated functions to scale synaptic strength, modulating synaptic plasticity (Perea et al., 2009; Eroglu and Barres, 2010; Araque et al., 2014). Furthermore, emerging evidence demonstrates the contribution of astrocytes to physiological behaviors, such as breathing, feeding and sleeping (Halassa et al., 2009; Gourine et al., 2010; Kim et al., 2014). Though some studies have begun paying attention to the role of astrocytes in learning and memory, including spatial learning and working memory (Pannasch and Rouach, 2013; Oliveira et al., 2015), little is known about the influence of astrocytes in associative memory and the mechanisms involved in it.

Fear conditioning, a classic model of associative fear learning, is well known to study the process of associative memory (Johansen et al., 2011; LeDoux, 2014; Yates, 2014). In this behavioral paradigm, animals learn to associate a neutral conditioned stimulus (CS, often a tone or context) with an aversive unconditioned stimulus (US, usually an electric footshock), forming a conditioned fear memory. Fear conditioning involves several stages of associative memory, including memory acquisition (the initial learning of CS-US association), consolidation (the transition of newly acquired memory into stable memory, known as short-term memory to long-term memory) and reconsolidation (the further processing of formed memory; McGaugh, 1966). Synaptic plasticity in the basolateral amygdala (BLA), composed of lateral amygdala (LA) and basal amygdala (BA), is critical for the conditioned fear memory formation, where CS-US inputs are integrated in LA and then memory is stored in BA (Rogan et al., 1997; Herry and Johansen, 2014; Bocchio et al., 2017). Lesion and functional inactivation of BLA are sufficient to blocking the formation of conditioned fear memory, associating with interfering synaptic plasticity (Rodrigues et al., 2004; Maren, 2005). Recent evidence indicate that astrocytic processes display dynamic structural changes with dendritic spines in response to synaptic activity and whisker stimulation, suggesting astrocytes undergo activity-dependent plasticity (Genoud et al., 2006; Lushnikova et al., 2009; Bernardinelli et al., 2014). However, it remains unclear whether and how astrocyte structural plasticity changed during conditioned fear memory.

Ras-related C3 botulinum toxin substrate 1 (Rac1), a member of small Rho GTPase family, is a pivotal protein in regulation of cytoskeletal remodeling (Jaffe and Hall, 2005). Rac1 acts between an inactive GDP-bound state and an active GTP-bound state, which is activated by guanine nucleotide exchange factors (GEFs). Several studies have been demonstrated that Rac1 activity and its downstream effectors modulate synaptic structural plasticity, as well as astrocytic morphogenesis (Heasman and Ridley, 2008; Rodnight and Gottfried, 2013; Zeug et al., 2017). Activation of Rac1 or GEF Tiam1 induces polarized protrusional outgrowth and increased cell area in primary astrocytes (Ellenbroek et al., 2012; Posada-Duque et al., 2015), whereas ablation or blockade of Rac1 activity reduce astrocyte stellations and process length in the cerebellum (Lippman et al., 2008; Racchetti et al., 2012). Meanwhile, the involvement of Rac1 in fear memory has also been reported. Fear conditioning induces Rac translocation and activation in hippocampus, and extinction of contextual fear memory suppresses Rac1 activation (Martinez et al., 2007). The activation of cerebral Rac1 before training enhances fear memory (Diana et al., 2007), while inhibition of Rac1 activity disrupts the acquisition, consolidation and reconsolidation of auditory fear memory (Wu et al., 2014; Gao et al., 2015). However, the role of astrocytic Rac1 in learning and memory is largely unknown.

## Materials and Methods

### Animals

Rac1^flox/flox^ mice were obtained from Jackson Laboratories (Stock number: 005550). C57BL/6J mice were purchased from Slaccas Lab Animal Ltd. All the mice were housed 2–4 per cage with access to food and water *ad libitum* at 22°C ± 2°C, and kept under a 12 h light/dark cycle (light off at 8:30 AM; light on at 8:30 PM). Eight to twelve weeks old male mice were used for behavioral experiments. All animal experiments were strictly in accordance with the National Institutes of Health Guide for the Care and Use of Laboratory Animals and were approved by Animal Care and Use Committee of Shanghai Medical College, Fudan University.

### Virus Construct and Packaging

Plasmids encoding Rac1 constitutive activated mutant (Rac1-CA) and dominant negative mutant (Rac1-DN) were as described previously (Long et al., 2013). Plasmids encoding photoactivatable Rac1 (Rac1-PA) and its photoinactive mutant Rac1-C450A were from Addgene (Plasmid # 22027 and # 22028). Lck sequences were synthesized in Sanggon. Rac1-CA and Rac1-DN were cloned into pAAV-hGFAP-mCherry as previously described (Tao et al., 2015); Rac1-PA, Rac1-C450A and LCK-mCherry were cloned into pAAV-mGFAP-HA-rM3D (Gs)-IRES-mCitrine (Addgene Plasmid # 50472). AAV_2/8_ was packaged at Obio Technology (Shanghai) Corp. Ltd., and the final viral titer was in the range of 1.0–2.0 × 10^13^ vg/ml. All AAV related hazards were sterilized by autoclaving or ultraviolet radiation for more than 20 min.

### Fluorocitrate (FC) Preparation

The solution of FC was prepared as described previously (Paulsen et al., 1987). Briefly, 8 mg of the barium salt of DL-fluorocitric acid (Sigma) was dissolved in 0.1 M HCl, precipitated by the addition of 2–3 drops of 0.1 M Na_2_SO_4_. Two microliters 0.1 M Na_2_HPO_4_ were added, and centrifuged at 1000 *g* for 5 min. The supernatant was diluted in 0.9% NaCl (saline) at a final concentration of 1 mM (pH 7.4). For intracerebroventricular injection, the dose of FC was 1 nmol. For intra-BLA injection, the dose of FC was 0.5 nmol for each side of bilateral BLA.

### Rac1 Activity Assay

Relative levels of GTP-bound Rac1 were determined by Rac1 Activation Assay Biochem Kit™ (Cytoskeleton) according to manufacturer’s procedure. Briefly, BLA were isolated and homogenized in IP lysis buffer (Beyotime Biotechnology) containing phosphotransferase inhibitor. Lysates were precipitated with PAK-PBD affinity beads for 4 h at 4°C, and then bound Rac1 protein was eluted from pelleted beads. Activated Rac1-mCherry and total Rac1-mCherry were examined by Western Blot with mCherry antibody (1:1000, Rockland), and endogenous activated and total Rac1 were detected with a Rac1 specific antibody (1:2000, BD Transduction Laboratories). For p-Cofilin and p-ERK measurement, cultured astrocytes expressing PA-Rac1 were irradiated for 5 min at 473 nm wavelength (6 mV), then lysed immediately with cold lysis buffer for Western Blot. All procedures were done at dark room to avoid unspecific light-activation of Rac1. For detecting Cofilin and ERK, antibody were used as follows: Cofilin (1:1000, CST), p-Cofilin (1:500, CST), ERK (1:2000, CST), p-ERK (1:2000, CST). Intensities of the detected bands in Western Blots were quantified in ImageJ software.

### Immunofluorescence

Immunofluorescence staining on mouse brain section was performed as previously described (Tao et al., 2015). In brief, mice anesthetized by 10% chloral hydrate were perfused intracardiacally with 30 ml normal saline, followed by 4% paraformaldehyde (4% PFA). Then the brains were quickly removed and post-fixed for about 12 h in the 4% PFA and further dehydrated in 30% PB-buffered sucrose solution for 36 h at 4°C. Brain slices were sectioned at 30 μm or 40 μm by a cryostat (Leica) and washed in PBS, blocked in the buffer (10% donkey serum and 0.2% Triton X-100 in PBS) for 90 min, and then incubated in primary antibody diluted in the blocking buffer overnight at 4°C. After being washed with PBS, brain slices were incubated in the secondary antibody diluted in the PBS for 2 h at room temperature, and washed with PBS. The slices were finally mounted with anti-quenching mounting medium (Thermo Fisher Scientific). The primary and secondary antibodies used for immunostaining were as follows: Rabbit anti-GFAP (1:2000, DAKO), Donkey anti-Rabbit 647 (1:1000, Jackson ImmunoResearch), Donkey anti-Rabbit 488 (1:1000, Jackson ImmunoResearch); 4′,6-diamidino-2-phenylindole (DAPI, 1:10,000, Sigma-Aldrich).

### Astrocyte Volume Measurement

All images were acquired using a Nikon laser-scanning confocal microscope (AIR-MP, Nikon) with a 25× water immersion objective with a 3× digital zoom factor. Acquisition settings were as follows: 1024 × 1024 frame size, 12-bit image resolution, a 0.5 μm step size, and Z-stacks were 30 μm. Three random sections in BLA were picked up for imaging, and then three-dimensional (3D) images were reconstructed and analyzed with Nikon imaging software (NIS-Elements). Single and non-overlap astrocyte with clear boundary was cropped for volume measurement following manual threshold setting. Measurement was performed in an unbiased manner, blind to the experimenter.

### Primary Astrocyte Culture and Imaging

Primary astrocyte culture was performed as described previously (Ferron et al., 2011). Briefly, isolated adult hippocampus tissues were transferred to DMEM/F12 medium (1:1 v/v; Life Technologies) containing 0.3% papain and incubated for 30 min at 37°C, then rinsed and triturated to a single-cell suspension. The isolated cells were re-suspended and seeded onto matrigel-treated (BD Transduction Laboratories) glass slides in 24 wells in astrocyte medium (DMEM/F12 medium containing 0.6% glucose, 5.2 ng/ml sodium selenite, 0.025 mg/ml insulin and 0.1 mg/ml transferrin, supplemented with 10% FBS). Cells were maintained at 37°C with 5% CO_2_ for 6 days and then shaken at 100 rotations/min for 3 h at 22°C to dissociate proliferating cells, oligodendrocyte progenitors, and neurons. For primary astrocyte imaging, AAV were added to astrocyte medium and incubated for 48 h, then astrocytes expressing mCherry were locally irradiated at 405 nm for 5 min. DIC images of cells before and after irradiation were adopted for analysis.

### Stereotactic Surgery

For viral microinjection, the anesthetized mice were positioned on a stereotaxic apparatus (Stoelting Co.) with the injection syringe of 34 gauge tips (Hamilton Bonaduz AG) aimed at BLA. The intended stereotaxic coordinates were: anterior-posterior (AP), −1.5 mm; medial-lateral (ML), ±3.4 mm; dorsal-ventral (DV), −4.8 mm, referenced to Bregma. 0.5 μl of AAV was infused into BLA at a rate of 0.1 μl/min. The needle was left in place for additional 5 min. For cannula implantation, mice were implanted with cannulas (26 gauge pedestal guide cannula, Plastics One) according to the following coordinates: lateral ventricle (ML: ±0.8 mm, DV: −2.3 mm), BLA (AP: −1.7 mm, ML: ±3.3 mm, DV: −3.7 mm). Optic fibers were implanted aimed at BLA (AP: −1.5 mm, ML: ±3.4 mm, DV: −4.6 mm). Cannulas and optic fibers were inserted and fixed to the skull with dental cement. All cannula placements and the virus expression in BLA were verified after the behavioral assays, and the animals with misplaced cannule implant or virus expression were excluded from subsequent analyses.

### Fear Conditioning

Two conditioning protocols were introduced (Li et al., 2009). For a single CS-US pairing paradigm, mice were placed in the conditioning chamber (MED Associates), and presented with one tone (CS, 2800 Hz, 85 dB, 30 s) that co-terminated with an electric footshock (US, 0.70 mA, 2 s). Alternatively, in the five CS-US pairings paradigm, mice received five tone-footshock pairing trials (CS, 2800 Hz, 85 dB, 30 s; US, 0.70 mA, 1 s) with 2-min intertrial interval, and freezing behavior was measured during each tone presentation. Then mice were conducted context test in 24 h and cue test in 48 h after conditioning. For contextual fear memory, mice were placed in the conditioning chamber for 5 min. For cued fear memory, mice were placed in a novel chamber for 3 min (Pre-cue) and then given 3 min tone (Cue). The freezing percentage was automatically analyzed by software, other than in light stimulation experiment, freezing behavior in five trials was scored by manual.

### *In Vivo* Photoactivation of Rac1

Mice were intra-BLA implanted with optic fiber (diameter, 200 μm; N.A., 0.37) after AAV injection. Before behavioral paradigm, optic fiber were attached to a 473-nm blue laser diode, and a continuous light with an output of 15 mV at the tip of fiber was generated. The 473-nm light was turned on for the duration of the fear conditioning.

### Statistical Analysis

Data were represented as means ± SEM and statistical analysis was performed by SigmaPlot 12.3. Behavioral data were analyzed by *t*-test or two-way repeated measures ANOVA followed by Bonferroni *post hoc* test. For astrocyte volume measurement, data were analyzed by one-way ANOVA on ranks. For Western Blot assay, data were analyzed by *t*-test. *P* < 0.05 is defined as statistically significant.

## Results

### Astrocyte Activity Is Required for the Acquisition of Conditioned Fear Memory

To determine whether astrocytes are involved in the conditioned fear memory, we investigated the effects of FC, an astroglial metabolism inhibitor to block the function of astrocytes (Fonnum et al., 1997), on the formation of fear memory. We performed cannula implantation on C57/BL6 mice. After an intracerebroventricular (i.c.v.) injection with 1 nmol FC, mice were conditioned by presenting a 30-s auditory tone (CS) that co-terminated with 2-s footshock (US). One day after conditioning, mice were re-exposed to the conditioning chamber in the absence of CS to evaluate the contextual fear memory, and exposed to novel context with the same tone in the training at 48 h to test the cued fear memory (Supplementary Figure S1A). Fluorocitrate treatment decreased freezing levels in both contextual (*t*-test; *t*_(16)_ = 2.268, *p* = 0.038) and cued fear memory (two-way RM ANOVA, *F*_treatment × session(1,16)_ = 8.438, *p* = 0.010) tests (Supplementary Figures S1B,C). We then examined the role of astrocytes in the acquisition of fear memory. Following an i.c.v. injection of FC, mice were conditioned with five paired CS (30-s tone)-US (1-s footshock) trials with 2-min intervals (Supplementary Figure S1D). Fluorocitrate treatment attenuated the fear memory acquisition and significantly decreased freezing was observed in 4th trial (Supplementary Figure S1E, two-way RM ANOVA, *F*_treatment(1,12)_ = 4.988, *p* = 0.045; 4th trial, *p* = 0.038).

Next, we investigated whether astrocytes in amygdala are required for fear memory acquisition. In this experiment, mice were intra-amygdala injected with FC and then conditioned by five CS-US pairing trials (Supplementary Figure S1F). Fluorocitrate treatment apparently made mice learn at a slower rate, and show less freezing levels (Supplementary Figure S1G, two-way RM ANOVA, *F*_treatment (1,28)_ = 5.030, *p* = 0.033; 4th trial, *p* = 0.021, 5th trial, *p* = 0.003). However, blocking the function of astrocytes did not affect the consolidation of fear memory, since FC treatment did not change freezing levels in both contextual and cued fear memory tests when injected immediately after conditioned with a single or five CS-US pairing paradigm. We also test anxiety, depression-like behaviors and shock reactivity on mice, FC treatment did not affect locomotor activity in open field, and the time spent in all areas of elevated plus maze (EPM), as well as locomotion to footshock (Supplementary Figure S2). Taken together, these results indicate that astrocyte activity in BLA is required for the acquisition of fear memory.

### Astrocytes in BLA Display Reduced Volume and Decreased Rac1 Activity Following Fear Conditioning

**Figure 1:**
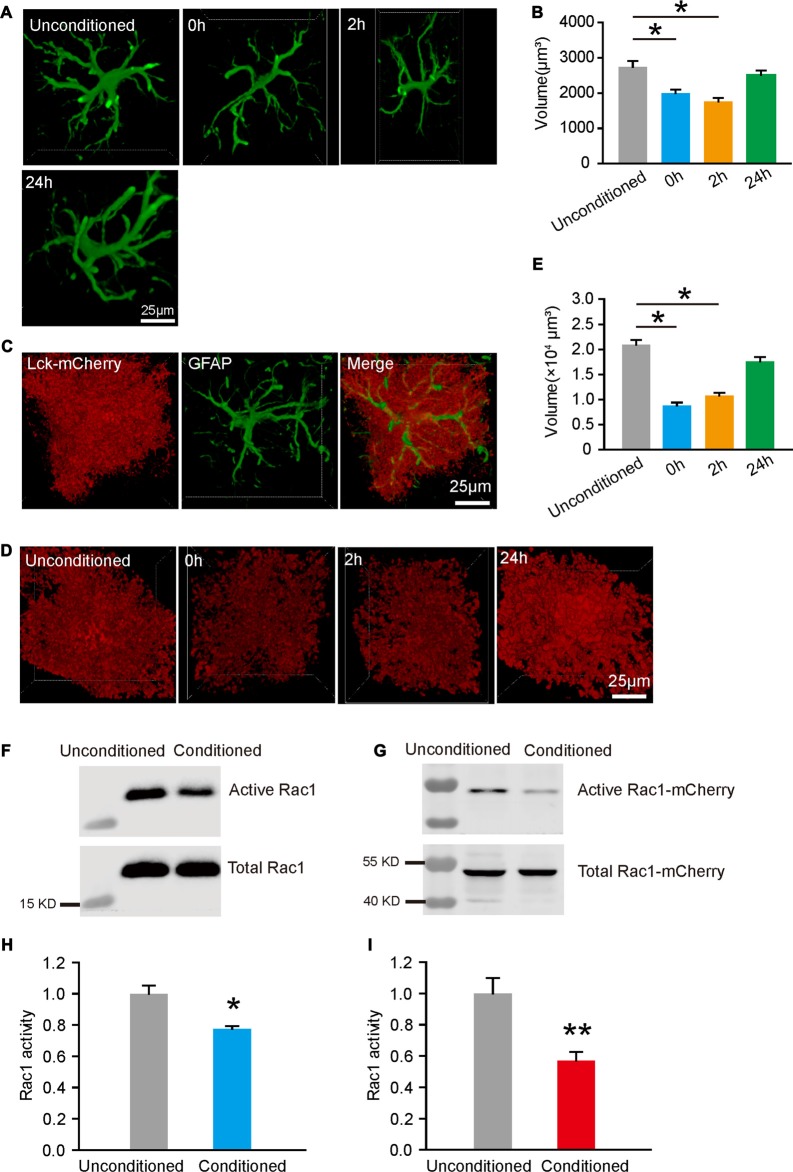
Fear conditioning induced reduced astrocytic volume and Rac1 activity in basolateral amygdala (BLA). **(A)** Representative images of 3D reconstructed astrocytes at different time-points following fear conditioning, based on GFAP staining. The cube in white line was automatic generated by software. **(B)** Fear conditioning induced changes in astrocytic volume at different time-points following fear conditioning (*n* = 80–100 cells from 3 mice per group). **(C)** A representative image of Lck-mCherry and GFAP labeled cells. **(D)** Representative 3D reconstructed images of astrocytes that labeled with Lck-mcherry at different time-points following fear conditioning. **(E)** Astrocytic volume was reduced after fear conditioning (*n* = 60–90 cells from 3 mice per group). **(F,G)** GTP-bound Rac1in the BLA **(F)** and astrocytes **(G)** was enrich by binding with PAK-PBD affinity beads and analyzed on Western Blots. **(H,I)** Fear conditioning decreased Rac1 activity in the BLA **(H)** and astrocytes **(I)** (*n* = 10 per group). *p < 0.05, **p < 0.01. Scale bar, 25 μm.

To determine how astrocytes are involved in fear memory acquisition, we detected the quantifiable alterations in the morphology of astroglial cells following fear conditioning. Immediately (0 h), 2 h or 24 h after mice were conditioned with five paired CS-US trials, brain sections were prepared. Astrocytes in BLA were imaged and single astrocyte was 3D reconstructed for volume measurement, based on GFAP immunostaining. We found that astrocyte exhibited reduced volume at 0 h and 2 h, and recovered at 24 h after conditioning (Figures 1A,B, one-way ANOVA on Ranks, *H*_(3)_ = 33.301, *p* < 0.001). Given that GFAP represents only a minor fraction of overall volume and not located in peripheral processes of an astrocyte, we infected astroglial cells in BLA with AAV expressing a membrane-associated mCherry (Lck-mCherry) under the control of *GFAP* promoter (Supplementary Figures S3A–C). As shown in 3D reconstructed images (Figure 1C), in addition to targeting to the plasma membrane and labeling the peripheral process of astrocyte, Lck-mCherry-expressing cells well colocalizated with GFAP-positive cells. Similarly, Lck-mCherry labeled astrocytes also displayed reduced volume at 0 h and 2 h, and returned to normal at 24 h (Figures 1D,E, one-way ANOVA on Ranks, *H*_(3)_ = 120.454, *p* < 0.001). The transient changes in volume suggest that astrocytes in BLA undergo structural plasticity following fear conditioning.

It was reported previously that Rac1 is associated with astrocytic structural changes, thus we tested whether fear conditioning would regulate astrocytic Rac1 activity. C57/BL6 mice were bilaterally intra-BLA injected AAV expressing Rac1-WT-mCherry under *GFAP* promoter. Activated Rac1, which bound to GTP, was pull-down by PAK-PBD affinity beads. The result showed that the total active Rac1 detected by Rac1 antibody was significantly decreased after conditioned with five paired CS-US trials. Moreover, using mCherry antibody to detect astrocytic Rac1 activity, we found that the amount of active Rac1 in astrocytes of BLA was down-regulated, compared with unconditioned group (Figures 1F,H, *t*-test, *t*_(10)_ = 3.805, *p* = 0.012; Figures 1G,I, *t*-test, *t*_(10)_ = 2.478, *p* = 0.002). We also tested Rac1 activity at 24 h following fear conditioning, found that the activity of astrocytic Rac1 was no significant difference between the conditioned and unconditioned group (Supplementary Figures S3D–G). These data reveal that fear conditioning induced a reduction in volume and suppression of Rac1 activity in BLA astrocytes.

### Astrocytic Rac1 in BLA Regulates the Acquisition of Fear Memory

**Figure 2:**
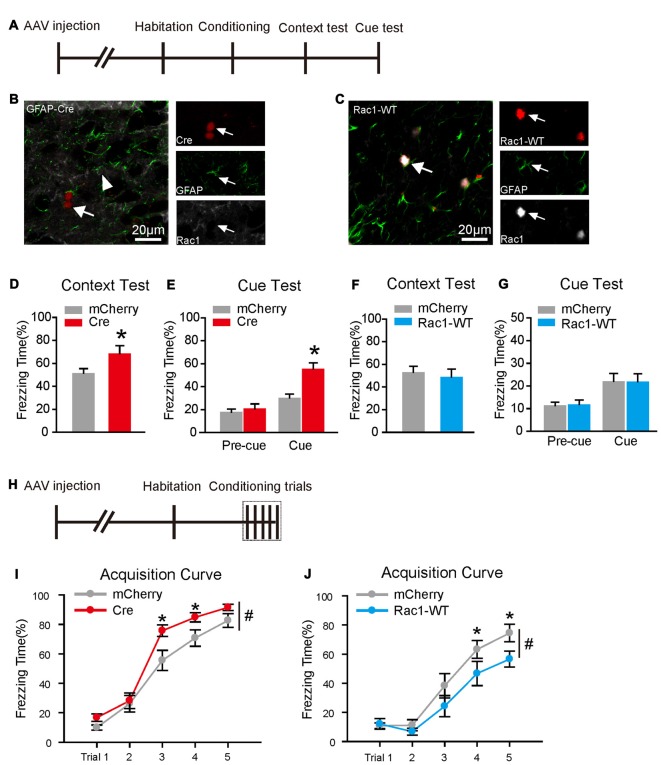
Astrocytic Rac1 in BLA regulated fear memory acquisition. **(A)** Experimental design. A single CS-US paired conditioning trial was carried out in Rac1^flox/flox^ mice infected with AAV expressing Cre recombinase and mCherry, or C57 mice infected with Rac1-WT and mCherry. **(B)** A representative image of Rac1 knockout in astrocyte. White arrows indicate that Rac1 is absent in Cre expressing astrocytes, while strongly expressed in non-Cre Cre expressing astrocytes. **(C)** Rac1 was strongly expressed in Rac1-WT expressing asrocytes. **(D,E)** Conditional knockout of astrocytic Rac1 in the BLA before the single CS-US paired conditioning trial increased freezing levels in contextual **(D)** and cued **(E)** memory tests (mCherry: *n* = 12, Cre: *n* = 8). **(F,G)** Overexpression of Rac1 in BLA astrocytes had no effect on contextual **(F)** or cued **(G)** memory tests (mCherry: *n* = 9, Rac1-WT: *n* = 9). **(H)** Experimental design. Five CS-US paired conditioning trials was carried out in Rac1^flox/flox^ imce infected with AAV expressing Cre recombinase and mCherry, or C57 mice infected with Rac1-WT and mCherry. **(I)** Conditional knockout of astrocytic Rac1 in the BLA improved fear memory acquisition (mCherry: *n* = 17, Cre: *n* = 16). **(J)** Overexpression of astrocytic Rac1 in the BLA attenuated fear memory acquisition (mCherry: *n* = 13, Rac1-WT: *n* = 12). *p < 0.05, ^#^*p* < 0.05. Scale bar, 20 μm.

To assess whether astrocytic Rac1 is involved in fear memory, we examined the consequence of Rac1 knockout or overexpression on conditioned fear memory. Rac1^flox/flox^ mice were bilaterally intra-BLA injected with AAV expressing Cre recombinase or mCherry under *GFAP* promotor (Figures 2A,B). Ablation of astrocytic Rac1 in BLA significantly increased freezing levels in both contextual and cued fear memory tests (Figure 2D, *t*-test, *t*_(18)_ = −2.298, *p* = 0.034; Figure 2E, two-way RM ANOVA, *F*_treatment × session(1,18)_ = 12.082, *p* = 0.003). However, overexpression of astrocytic Rac1 in BLA did not affect contextual and cued fear memories (Figures 2C,F,G).

We further examined whether astrocytic Rac1 is involved in fear memory acquisition. During the five CS-US parings of fear conditioning (Figure 2H), we found that ablating Rac1 in BLA astrocytes led mice to learn at a faster rate (Figure 2I, two-way RM ANOVA, *F*_treatment (1,31)_ = 4.785, *p* = 0.036; 3rd trial, *p* = 0.036, 4th trial, *p* = 0.030). Conversely, overexpression of astrocytic Rac1 attenuated the rate of fear learning (Figure 2J, two-way RM ANOVA, *F*_treatment (1,23)_ = 4.388, *p* = 0.047; 4th trial, *p* = 0.045; 5th trial, *p* = 0.031). In agreement with FC treatment (Data not shown), Rac1 knockout or overexpression had no significant effects on long-term memory following five CS-US pairing trials (Supplementary Figure S4), and we speculate that it might be due to a ceiling effect, since all group mice showed a considerable level of freezing in trial 5 during conditioning. Therefore, these data indicate that BLA astrocytic Rac1 negatively regulates fear memory acquisition.

### Activation of Rac1 in BLA Astrocytes Attenuates the Acquisition of Fear Memory

**Figure 3:**
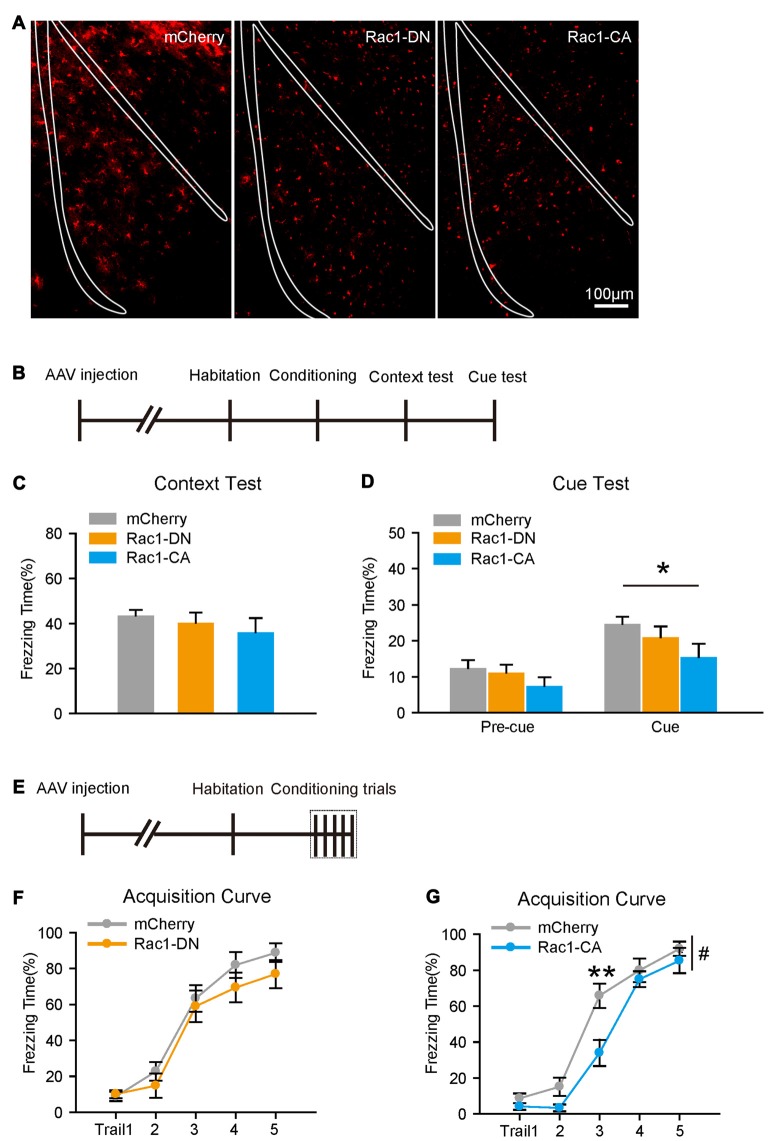
Constitutive activation of astrocytic Rac1 in the BLA impaired fear memory acquisition. **(A)** Representative images of BLA infected by AAV expressing mCherry, Rac1-DN or Rac1-CA. **(B)** Experimental design. A single CS-US paired conditioning trial. **(C,D)** Overexpression of Rac1-CA, but not Rac1-DN, in the BLA decreased freezing levels in cued memory test (mCherry: *n* = 12, Rac1-DN: *n* = 13, Rac1-CA: *n* = 11). **(E)** Experimental design. Five CS-US paired conditioning trials. **(F,G)** Overexpression of astrocytic Rac1-CA, but not Rac1-DN, in the BLA impaired fear memory acquisition (mCherry: *n* = 11–12, Rac1-CA: *n* = 12, Rac1 DN: *n* = 10). *p < 0.05, **p < 0.01, ^#^*p* < 0.05. Scale bar, 100 μm.

Given that Rac1 usually acts as an inactive or active form, we further investigated whether the activity of astrocytic Rac1 regulates conditioned fear memory. C57/BL6 mice were bilaterally intra-BLA injected with AAV expressing a dominant-negative (T17N) mutant of Rac1 (Rac1-DN) or a constitutively active mutant (Q61L) of Rac1 (Rac1-CA) under *GFAP* promotor (Figure 3A). The expression of Rac1-DN showed no significant effects on either contextual or cued fear memory. While constitutively activated Rac1 did not reduce freezing levels in contextual fear memory test, but attenuated cued fear memory (Figures 3B–D, two-way RM ANOVA, *F*_treatment (2,33)_ = 3.844, *p* = 0.031), indicating that activation of astrocytic Rac1 in BLA inhibits the formation of auditory fear memory.

We then tested whether Rac1 activity would affect the acquisition of fear memory. Constitutive activation of Rac1 led mice to learn at a slower rate, whereas the expression of Rac1-DN had no effect on conditional training (Figures 3E–G; Figure 3G, two-way RM ANOVA, *F*_treatment (1,22)_ = 6.093, *p* = 0.022, 3rd trial, *p* < 0.001). In the long-term memory test, constitutive activation of Rac1 impaired contextual fear memory, while the expression of Rac1-DN had no significant effect on either contextual or cued fear memory (Supplementary Figures S5A–F). These results support the notion that the activation of Rac1 in BLA astrocytes inhibits the acquisition of fear memory, while the downregulation of Rac1 activity in astrocytes facilities fear memory acquisition.

### Photo-Stimulation Induced Activation of Rac1 in Astrocytes Induces Morphological Changes and Attenuates the Acquisition of Fear Memory

Rac1 and other small G proteins, such as RhoA and CDC42, are members of the Rho GTPase family, and the prolonged overexpression of constitutive active form of Rac1 might cause compensational response. Therefore, we introduced photoactivatable Rac1 (Rac1-PA), which Rac1-CA fused with a photoreacitve light oxygen voltage (LOV) domain (Wu et al., 2009). In the presence of a 405–473 nm light stimulation, Rac1-PA is activated rapidly, thus enable us to investigate the morphological and behavioral consequences of activation of Rac1 in astrocytes on a real time scale.

**Figure 4:**
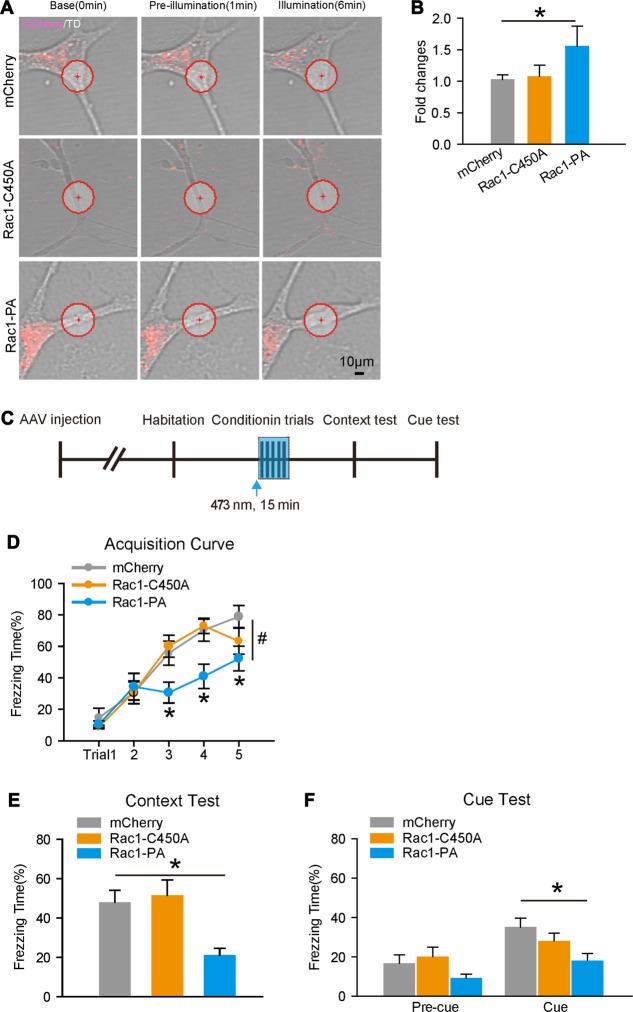
Temporal activation of Rac1 induced structural changes in cultured astrocytes and impaired fear memory acquisition. **(A)** Representative images of primary cultured astrocytes in different time-points following light stimulation. Red circles represent the spots of light stimulation. Light activation of Rac1-PA in an astrocytic process induced the generation of protrusions and increased cell area. **(B)** Phtoactivation of Rac1 significantly increased cell surface areas in cultured astrocytes (*n* = 6 per group). **(C)** Experimental design. Five CS-US paired conditioning trials with 473 nm light simulation in the BLA infected by AAV expressing Rac1-PA, Rac1-C450A, or mCherry during conditioning. **(D–F)** Light activation of Rac1-PA in the BLA attenuated fear memory acquisition. Similarly, light simulation of Rac1-PA impaired both contextual and cued fear memories (mCherry: *n* = 14, Rac1-C450A: *n* = 12, Rac1-PA: *n* = 13). *p < 0.05, ^#^*p* < 0.05. Scale bar, 10 μm.

We first examined whether the activation of Rac1 would be associated with morphological changes in cultured astrocytes, since fear conditioning induced both downregulation of Rac1 activity and morphological alterations in astrocytes. AAV encoding Rac1-PA or Rac1-C450A (Rac1 fused to light-insensitive LOV, C450A) under *GFAP* promotor was introduced into primary astrocytes. We found that light stimulation of Rac1-PA sufficiently activated its downstream effector, with increased level of p-Cofilin (Supplementary Figures S5G,H). Moreover, photoactivation of Rac1 induced local protrusions with increased surface area in Rac1-PA expressing astrocytes, but not Rac1-C450A astrocytes (Figures 4A,B), indicating that Rac1 activation results in astrocyte morphological changes.

Next, we investigated the effects of temporal activation of astrocytic Rac1 *in vivo* during conditioning on fear memory. C57/BL6 mice received intra-BLA injection of AAV expressing Rac1-PA, Rac1-C450A, or Lck-mCherry, and were fitted with fiber optic cables (Figure 4C). Consistent with results obtained with Rac1-CA, photoactivation of Rac1 during conditioning attenuated fear learning (Figure 4D, two-way RM ANOVA, *F*_treatment × session(2,36)_ = 2.654, *p* = 0.010, 3th trial, *p* = 0.026, 4th trial, *p* = 0.007, 5th trial, *p* = 0.016) and decreased freezing levels in both contextual and cued fear memory tests (Figure 4E, one-way ANOVA on Ranks, *H*_(2)_ = 12.797, *p* = 0.002; Figure 4F, two-way RM ANOVA, *F*_treatment (2,36)_ = 3.500, *p* = 0.041). Light stimulation of Rac1-C450A had no effects on conditioned fear memory (Figures 4D–F). These data thus confirm that the stimulation of Rac1 activity in astrocytes during conditioning could attenuate fear memory acquisition, and that fear conditioning-induced transient downregulation Rac1 activity in astrocytes facilitates the formation of conditioned fear memory.

## Discussion

Our results provide a fundamentally new insight to the basis by which astrocytes regulate the formation of conditioned fear memory. By pharmacological approach, we found astrocyte metabolic activity is required for fear memory acquisition. Fear conditioning induced astrocyte structural plasticity in the BLA, in parallel with the downregulation of Rac1 activity. Furthermore, overexpression of Rac1 or constitutively active Rac1 attenuated fear memory acquisition, suggesting downregulation of Rac1 activity in astrocytes facilitates the formation of fear memory. Indeed, overexpression of Rac1-PA in astrocytes, which allowed us to activate Rac1 in a real-time manner, significantly blocked the formation of both contextual and cued fear memories. In addition, transient activation of Rac1 induced morphological changes in primary astrocytes, providing an indicator of Rac1 activity associated with astrocyte structural plasticity. In conclusion, our study reveals suppression of Rac1 activity in astrocytes associated with structural plasticity is critical for fear memory formation.

It is well known that astrocytes could respond to neurotransmitters and release gliotransmitters to scale synaptic activity and plasticity in turn, suggesting they might directly regulate learning and memory (Pannasch and Rouach, 2013; Oliveira et al., 2015). Ablation of astrocytes or blockade of astrocytic glutamate uptake in the prefrontal cortex impair rat spatial memory (Bechtholt-Gompf et al., 2010; Lima et al., 2014). Knockout of astrocytic CB1 receptors induce long-term depression (LTD) in the hippocampus and deficiency in working memory (Han et al., 2012). Recently, studies have demonstrated that astrocyte-neuron lactate transports is required for long-term memory formation (Suzuki et al., 2011). Inhibition of lactate transports could efficiently prevent cocaine relapse, indicating astrocytes are possibly involved in associative memory (Boury-Jamot et al., 2016; Zhang et al., 2016). In the current behavioral paradigm, FC, a glial metabolism inhibitor, impairs the formation of conditioned fear memory, which mainly acts on the acquisition phase but leave memory consolidation intact and we found focal inhibition of astrocytes in the BLA is sufficient to attenuated fear memory acquisition. In agreement with our results, FC is previously reported decreasing synaptic transmission and damaging working memory (Filosa et al., 2009; Wang et al., 2009; Bonansco et al., 2011). These conform findings suggest that astrocytes could modulate associative memory, and their metabolism activity is one critical part when they modulate the formation of fear memory.

Another potential mechanism of regulating synaptic plasticity is the structural interplay between astrocytes and synapses. Astrocyte process-dendritic spine interactions is a critical additional elements underling experience-induced plasticity (Theodosis et al., 2008). Astrocyte processes exhibit rapidly extension and retraction to engage and disengage from dendritic spines, helping stable larger spines in hippocampus (Haber et al., 2006). Further studies reveal that astrocyte structural plasticity is neuronal activity dependent. Synaptic potentiation produces increased glial coverage of synapses in CA1 hippocampus (Lushnikova et al., 2009), and both synaptic activation and sensory stimulation could induce astrocytic process remodeling *in vitro* or *in vivo* (Bernardinelli et al., 2014; Medvedev et al., 2014; Perez-Alvarez et al., 2014). Moreover, it is reported that cocaine self-administration and extinction leads to reduced GFAP expression and volume in the nucleus accumbens core (Scofield et al., 2016), suggesting the entire of morphology in astrocytes might undergo plasticity. As to fear memory, one report showed that increased synapse lacking astrocyte appear in the amygdala during fear conditioning (Ostroff et al., 2014). In this study, we added more detailed temporal and spatial information on astrocyte morphological changes associated with learning induced circuitry plasticity. We found that fear conditioning could immediately induce the structural plasticity in astrocytes, which experience a reduced volume and then return to normal in 24 h following fear training, supporting our proposition that reduced astrocyte volume is in favor of fear memory formation.

Rac1 is a key regulator in cytoskeleton reorganization. Several reports have indicated that Rac1 activity is involved in dendritic remodeling, though a complicated mechanism might be associated with (Newey et al., 2005; Gordon-Weeks and Fournier, 2014). For example, activation of Rac1 promotes spine formation while blockade of Rac1 activation inhibits the stability of mature spines (Tashiro et al., 2000; Tashiro and Yuste, 2004). Rac1 activity also regulates astrocyte morphological features. Disruption of Rac1 activity leads to changes in kinds of astrocytic processes, as well as cell area and process length (Lippman et al., 2008; Racchetti et al., 2012; Posada-Duque et al., 2015). Here, we found that Rac1 activity is suppressed by fear conditioning, along with astrocyte structural changes, giving a hint for the relationship between them. Indeed, When Rac1 is activated in the local process in real time, protrusional outgrowth is generated in primary astrocytes. These results provide a possible explanation that fear conditioning induced the downregulation of Rac1 activity is associated with astrocyte structural plasticity.

The activity of Rac1 is also critical for regulating learning and memory. Activation of cerebral Rho GTPases, including Rac1, improves fear memory retention and spatial learning (Diana et al., 2007), whereas ablation of Rac1 in excitatory neurons in mouse forebrain causes deficits in working memory (Haditsch et al., 2009). Inhibition of Rac1 activity in the BLA could disrupt the consolidation and reconsolidation of auditory fear memory, while inhibition of Rac1 activity in the hippocampus impair the reconsolidation of contextual fear memory (Wu et al., 2014; Gao et al., 2015). Other studies report that inhibition of Rac1 activity in the hippocampus impairs extinction of contextual fear (Jiang et al., 2016) and activation of Rac1 activity promotes memory forgetting (Shuai et al., 2010; Liu et al., 2016). However, much attention are paid to the role of Rac1 in local brain regions or neuronal cells, the effects of astrocytic Rac1 on learning and memory are overlooked. Our study showed that ablation of Rac1 in astrocytes promotes fear memory acquisition, while overexpression or constitutive activation of astrocytic Rac1 impairs fear memory acquisition, suggesting suppression of Rac1 activity in astrocytes is necessary for fear memory formation. Inconsistently, Rac1 KO in astrocytes led to a significant promotion in fear memory acquisition, while Rac1-DN had no obvious effects. It is possibly that expression of exogenous Rac1-DN in astrocytes mainly inhibits endogenous Rac1 activitiy by competing for upstream activators. However, Rac1 KO totally abolishes Rac1 in astrocytes, which might have a stronger and more specific inhibited effect on Rac1 activity than Rac1-DN, leading to more obviously behavioral effect. Rac1-PA, a form of photoactivatable Rac1, could allow us to manipulate the Rac1 activity in real time both *in vitro* and *in vivo*. Recently, it has reported that light simulation of Rac1-PA in neurons successfully prevents the formation of a conditioned place preference to cocaine and could erase mouse acquired motor skills (Dietz et al., 2012; Hayashi-Takagi et al., 2015). Strikingly, temporal activation of Rac1 in astrocytes during the fear conditioning phase, obviously attenuates fear memory acquisition, as well as impairs both contextual and cued fear memories. This effect is more significant than we observed in constitutive activation of Rac1. Possibly, light simulation of Rac1-PA might avoid of compensatory responses caused by other Rho GTPases. Meanwhile, we found that Rac1-PA, which was mainly located in subplasma membrane (data not shown) due to fusing into LOV domain, might induce a stronger effect on Rac1 activity and astrocyte morphology. Thus, it provides another interpretation that photoactivation of Rac1 led to a more obvious behavioral outcome than Rac1-WT and Rac1-CA. Above all, we speculate that fear conditioning might suppress Rac1 activity to regulate astrocyte extension, thus facilitate the interaction between astrocyte and neuron, as well as the interaction between neurons to favor the memory formation.

Together, our data demonstrate that astrocyte activity is required for fear memory formation. Fear conditioning induces the downregulation of Rac1 activity and structural plasticity in astrocytes which facilitates the fear memory formation. To our knowledge, this study first reveals the behavioral consequences of astrocytic Rac1, providing a new insight into the role of astrocytes in learning and memory, but the downstream signaling pathways and the impact on synaptic plasticity induced by astrocytic Rac1 remain to be elucidated.

## Author Contributions

ZL, YT, XG and DC performed the experiments. LM, ZL and YT analyzed the data and wrote the manuscript. LM, ZL, FW and XL designed the study.

## Conflict of Interest Statement

The authors declare that the research was conducted in the absence of any commercial or financial relationships that could be construed as a potential conflict of interest.
