# Supplementary material for: Fear Conditioning Downregulates Rac1 Activity in the Basolateral Amygdala Astrocytes to Facilitate the Formation of Fear Memory
Source: Front Mol Neurosci. 2017 Nov 27;10:396. doi: 10.3389/fnmol.2017.00396 (PMC5712045; doi:10.3389/fnmol.2017.00396)
Supplement: Supplementary file 1 [file Presentation_1.pdf]

Supplementary Material

Fear Conditioning Downregulates Rac1 Activity in the Basolateral Amygdala Astrocytes to Facilitate the Formation of Fear Memory

Zhaohui Liao*, Yezheng Tao*, Xiaomu Guo, Deqin Cheng, Feifei Wang, Xing Liu, Lan Ma#

*** Co-first author**

# Correspondence: Lan Ma, lanma@fudan.edu.cn

Supplementary Figures

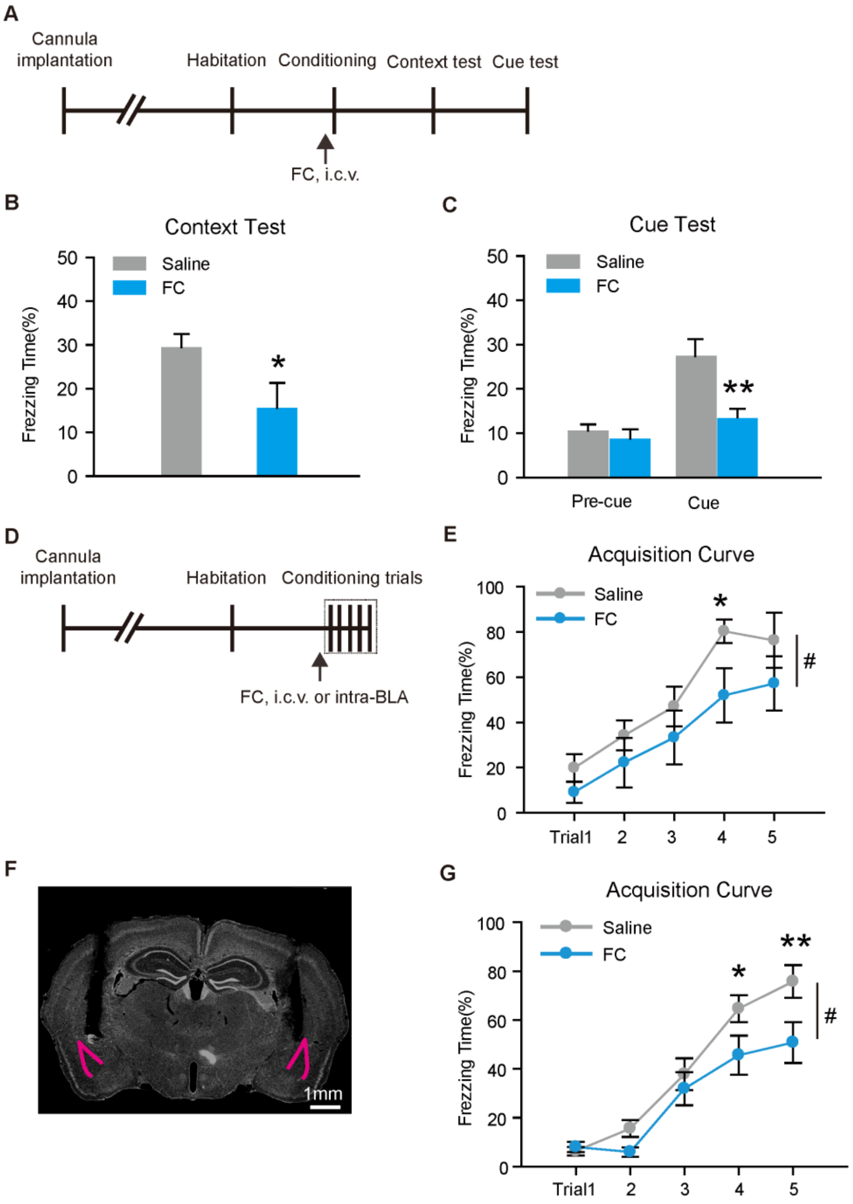

Figure S1. Fluorocitrate impaired the formation of conditioned fear memory. **(A)** Experimental design. An intracerebroventricular (i.c.v.) injection of fluorocitrate (1 nmol) before a single CS-US paired conditioning trial. FC, fluorocitrate. **(B, C)** Fluorocitrate decreased freezing levels in contextual (**B**) and cued (**C**) memory tests (Saline: n = 10, FC: n = 8). **(D)** Experimental design. Five CS-US paired conditioning trials were conducted. BLA, basolateral amygdala. **(E)** Intracerebroventricular injection of fluorocitrate attenuated fear memory acquisition and decreased freezing levels (Saline: n = 10, FC: n = 8). **(F)** A representative image of cannula implantation into BLA. **(G)** Intra-BLA injection of fluorocitrate attenuated fear memory acquisition and decreased freezing levels (Saline: n = 14, FC: n = 16). $^*p < 0.05$, $^{**}p < 0.01$, $\#p < 0.05$. Scale bar, 1 mm.

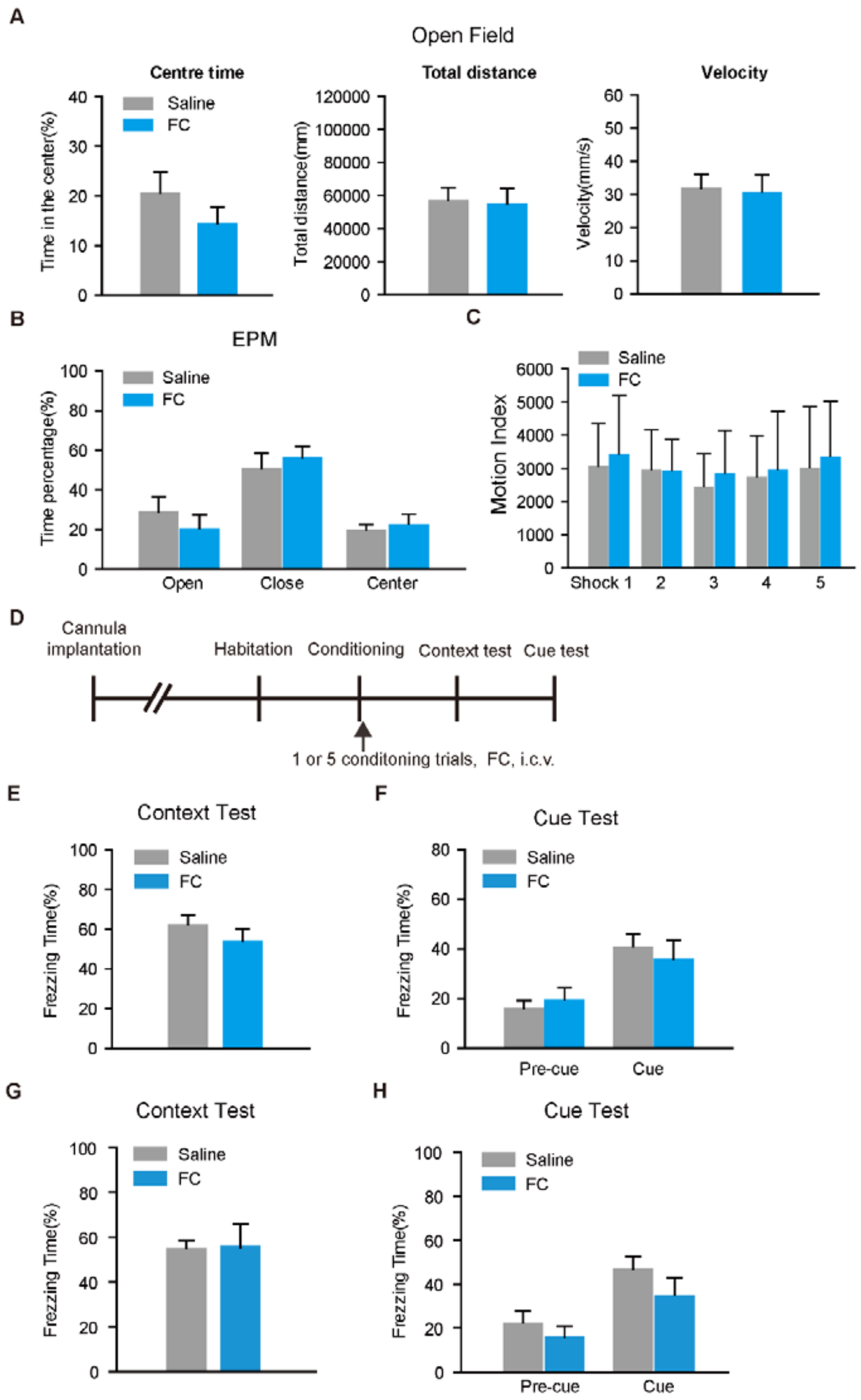

Figure S2. The effects of fluorocitrate on anxiety and depression-like behaviors, and fear memory consolidation. **(A)** Fluorocitrate did not change locomotor activity in open field test (*t*-test, $p > 0.05$). **(B)** Fluorocitrate did not affect the time spent in all areas of EPM (two-way ANOVA, $p = 0.481$). **(C)** Fluorocitrate did not significantly change motion sensitivity to each shock during fear conditioning (two-way RM ANOVA, $p > 0.05$). **(D-H)** Fluorocitrate had no significant effects on fear memory consolidation following a single CS-US training (**E, F**)or five CS-US paired trainings (**G, H**).

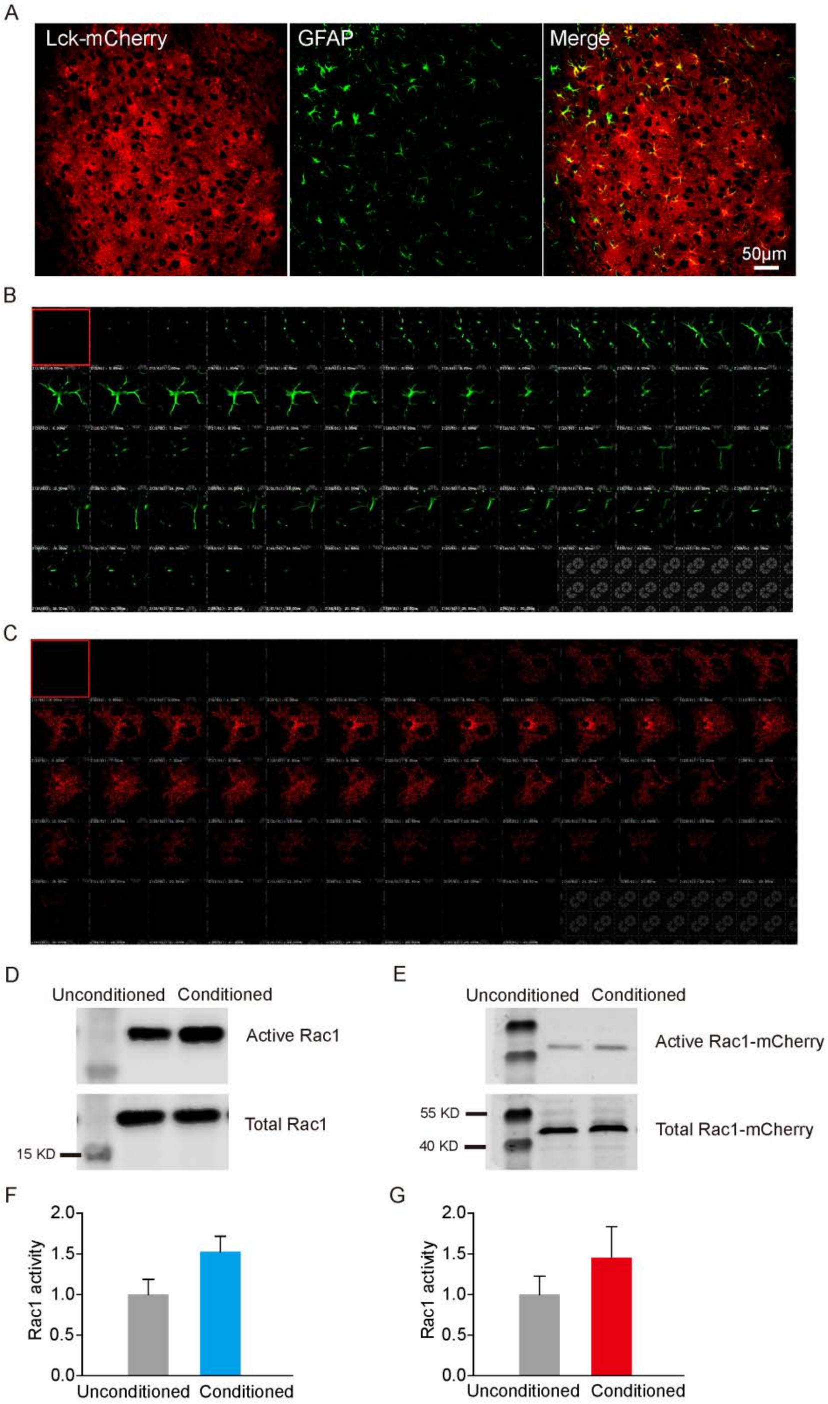

Figure S3. Fear conditioning induced reduced astrocytic volume and Rac1 activity in basolateral amygdala. **(A)** Colocalization of Lck-mCherry and GFAP in the BLA astrocytes. **(B, C)** A representative image of a multiple scanned astrocyte expressing GFAP (**B**) and Lck-mCherry (**C**). **(D-G)** Following fear conditioning at 24 h, both total (**D, F**) and astrocytic Rac1 activity (**E, G**) showed no significant difference to the unconditioned group (*t*-test, $p > 0.05$).

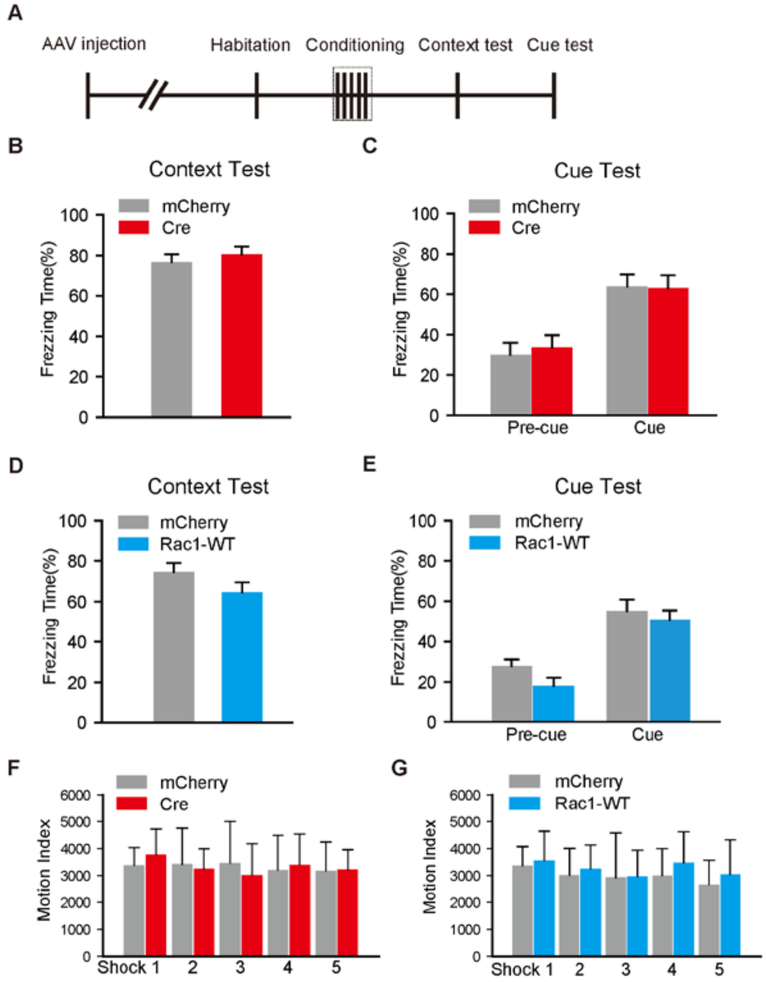

Figure S4. Rac1 knockout or overexpression in BLA astrocytes had no effect on long-term fear memory following five CS-US paired conditioning trials. **(A)** Experimental design. **(B, C)** Ablation of astrocytic Rac1 made no differences in contextual (B, *U*-test, $p = 0.439$) and cued fear memory (C, two-way RM ANOVA, $p = 0.564$). **(D, E)** Overexpression of astrocytic Rac1 also produced no effects on contextual (**D**, *t*-test, $p = 0.144$) and cued fear memory (**E**, two-way RM ANOVA, $p = 0.268$). **(F, G)** Knockout or overexpression of Rac1 did not change motion sensitivity to each shock during the conditioning trials (**F**, two-way RM ANOVA, $p = 0.320$; **G**, $p = 0.957$).

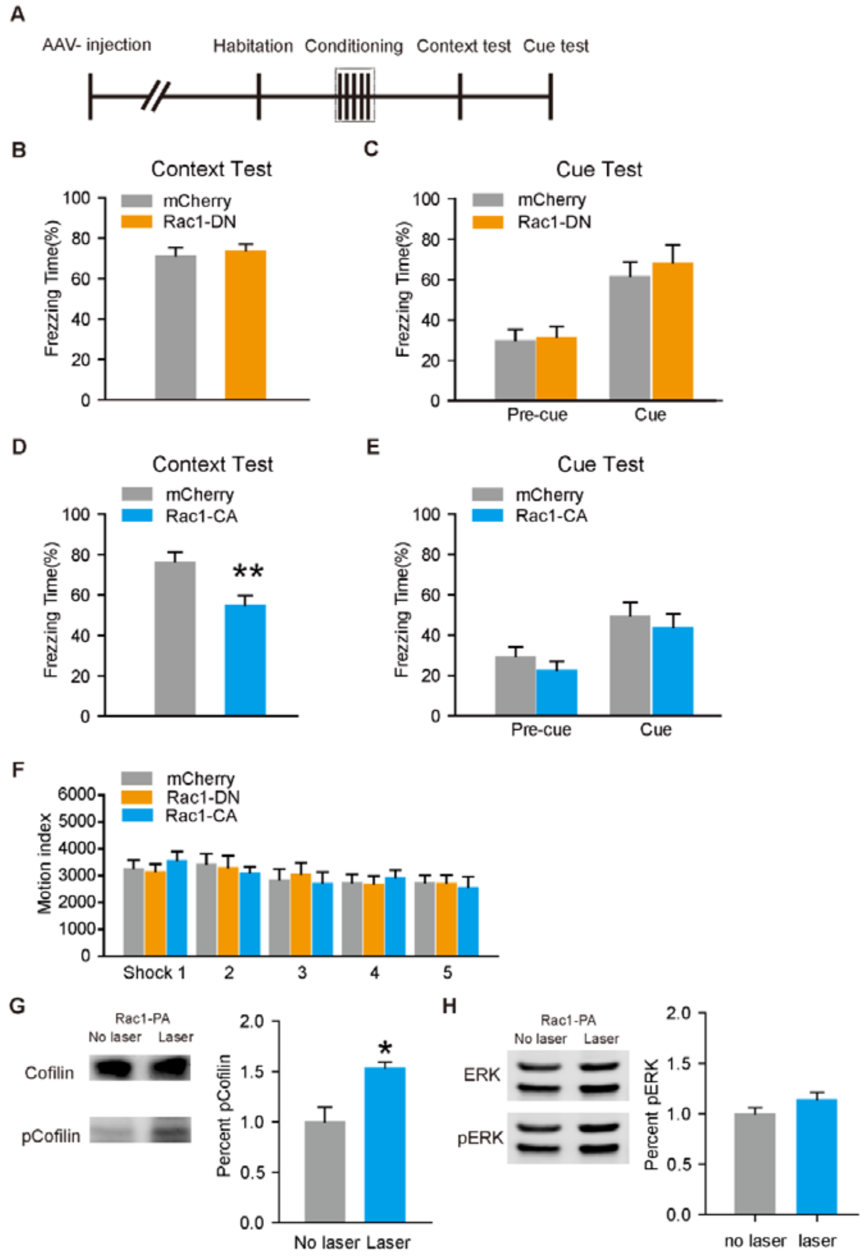

Figure S5. Constitutive activation of Rac1 in BLA astrocytes following five CS-US paired conditioning trials impaired contextual fear memory, while had no effects on cued fear memory. **(A)** Experimental design. **(B, C)** Overexpression of Rac1-DN in BLA astrocytes had no differences in contextual (**B**, *t*-test, $p = 0.621$) and cued fear memory (**C**, two-way RM ANOVE, $p = 0.341$). **(D, E)** Overexpression of Rac1-CA in BLA astrocytes impaired contextual fear memory (**D**, *t*-test, $p<0.01$), but produced no effects on cued fear memory (**E**, two-way RM ANOVA, $p = 0.909$). **(F)** Overexpression of Rac1-DN and Rac1-CA had no effects on motion sensitivity to each shock during fear conditioning trials. **(G and H)** Photoactivation of Rac1 significantly up-regulated p-Cofilin (**G**, *t*-test, $p = 0.026$) while had no effects on nonrelated proteins such as p-ERK (**H**). $*p < 0.05$, $**p < 0.01$.
